# Pit and Fissure Sealants—A Comprehensive Review

**DOI:** 10.3390/dj6020018

**Published:** 2018-06-12

**Authors:** Barbara Cvikl, Andreas Moritz, Katrin Bekes

**Affiliations:** 1 Department of Conservative Dentistry & Periodontology, School of Dentistry, Medical University of Vienna, A-1090 Vienna, Austria; andreas.moritz@meduniwien.ac.at; 2 Department of Pediatric Dentistry, School of Dentistry, Medical University of Vienna, A-1090 Vienna, Austria; katrin.bekes@meduniwien.ac.at

**Keywords:** caries, fissures, non-cavitated lesions, prophylaxis, sealing

## Abstract

Even in the 21st century, dental caries is considered a global burden, severely upsetting the health and quality of life of those affected. Apart from the usage of fluoride and regular oral hygiene, one of the most important prophylactic approaches against the occurrence of caries is the sealing of pits and fissures. However, the rapid progress of new materials and applications for sealing pits and fissures also raises new questions about their correct application. Recent literature on pit and fissure sealing, caries prevention, as well as caries risk assessment for both children and adults was reviewed. This report provides a general overview of pit and fissure sealing, the materials used for sealing occlusal surfaces, as well as indications and possible side effects. The conclusions are that sealing pit and fissures of primary and permanent teeth is an effective method for preventing and arresting caries. However, regular checkups must be conducted to avoid advanced tooth decay attributable to leakages in the sealing.

## 1. Caries Developments in Recent Decades

Dental caries reached a climax in the 19th and 20th centuries due to the increased availability of sugar for the general population of developed countries [1]. Only with the extensive use of fluorides in the 1970s was the rapid rise of the disease of dental hard tissue diminished [2]. Nevertheless, dental caries is one of the most common intraoral diseases, with serious consequences for both the individual patient and for the public in terms of medical, social, and economic concerns. The individual patient suffers from pain, dysfunction of the oral system, and reduced quality of life [3], while the general public must bear the cost of treatment and possible lost productivity of those affected.

Recent reports have confirmed an increase in caries on a worldwide scale, confirming its status as an important global oral health burden [4,5,6,7]. Unfortunately, caries predominantly attack the occlusal surfaces of premolars and molars during their eruption [8]. On the other hand, smooth surface caries have shown a significant decline, likely a result of increased worldwide access to fluoride [9,10]. In previous reports, an incomplete post-eruptive maturation [11,12,13] and the presence of narrow and deep fissures were blamed for the increased caries susceptibility of occlusal surfaces [14]. However, this theory has been questioned [8].

A more obvious explanation seems to be that dental plaque can mature undisturbed in the pits and fissures of teeth in eruption; as a result, enamel will be dissolved by the unimpeded repeated acid attacks [15]. This also explains why fluorides are not as effective in pits and in the fissure system as on smooth surfaces. Fluorides can effectively inhibit demineralization, promote remineralization, and also prevent acid formation by bacteria. However, they must act on a local level, which is not always possible with pit and fissures. A recently published Cochrane review reported a decrease of caries in 3.7% and 29% of children after two and nine years, respectively, when a resin-based sealant was placed in comparison to fluoride varnish applications [16]. This could be seen as an indirect suggestion for favoring resin-based sealing materials.

During tooth eruption, natural cleaning mechanisms through the tongue, lips, and cheeks during chewing and swallowing are absent [8]. The occlusal surfaces of erupting teeth are especially affected by the reduced ability of cleaning [17]. As a result, bacteria and food residues can accumulate in the pits and fissures, produce a biofilm, and lead to demineralization and caries [15]. An epidemiological study investigating data from 2011–2012 observed that the decrease in occlusal surface caries had not kept pace with the decrease in smooth surface caries [10]. The authors assumed that preventive interventions, such as the addition of fluoride to water and toothpastes as well as topical fluoride application, more effectively reduced caries on smooth surfaces than in pits and fissures [10].

## 2. Caries Prophylaxis

Indisputable approaches to preventing caries are regular oral hygiene with fluoride-containing toothpaste, a reduction in the intake of cariogenic food, as well as local and systemic fluoridation. For anatomically sensitive areas such as pits and fissures, additional approaches exist. The idea of sealing pits and fissures of occlusal surfaces had already been developed by the 1960s [18,19,20]. Sealing the surface creates a physical barrier that blocks the biofilm’s nutrition and, as a result, inhibits biofilm growth [21,22]. Hence the use of sealing materials is a simple physical problem solution as fluorides inhibit demineralization, promote remineralization, and prevent acid formation by plaque bacteria [23,24]. Nevertheless, numerous investigations have reported on the significant benefits for resin-based sealants in comparison with fluoride varnishes [25,26,27]. The efficacy of resin-based sealing materials is beyond question, having been proven in many studies [28,29]. One study even showed that a time-delayed application of the fissure sealant of about one year already led to a substantial increase in caries frequency [30]. However, its efficacy is dependent on a tight closure [21].

A conference paper of the American Academy of Pediatric Dentistry, Pediatric Restorative Dentistry Consensus Conference in 2002 strictly recommends the use of sealing materials in permanent molars in children and adolescents [10]. This advocacy is based on an analysis of nine randomized controlled trials on permanent molars with a follow-up period of two to three years. The incidence of caries showed a reduction of about 76% in occlusal caries [31,32,33,34,35,36,37]. Furthermore, a comparison of the efficacy of sealing materials and of fluoride varnishes was conducted with reference to three randomized controlled trials [32,34,38]. In addition, a reduction of about 73% of the incidence of occlusal caries in permanent molars after two to three years in the sealing groups was found in comparison to the fluoride varnish group.

Another study by Bravo et al. [27], which placed sealing materials on sound occlusal surfaces, reported on a caries incidence of only 27% in sealed surfaces compared to a caries incidence of about 77% in the unsealed control group, and about 56% in another control group using fluoride varnishes after nine years. A clinical study with 360 children and an observation period of 15 years showed a reduction in caries of 36% when all first molars were sealed and a reduction in caries of 54% when all posterior teeth were sealed [39]. Positive reports on the use of sealing materials were also confirmed by a systematic review of the Cochrane library [20]. The exploration of randomized and quasi-randomized controlled trials with a minimum period of 12 months, which compared the efficacy of sealing materials on occlusal and approximal surfaces with no sealing, resulted in an unambiguous recommendation: “the application of sealants is a recommended procedure to prevent or control caries” [20].

## 3. Materials for Sealing Pits and Fissures

Thus, the advantages of sealing materials in the field of caries prophylaxis are undisputed. However, the material most suitable for the indication of pit and fissure sealing is still under question. Resin-based sealants and glass ionomer sealants are most commonly used as sealing materials [10]. Although resin-based sealants are composed of urethane dimethacrylate (UDMA) or bisphenol A-glycidyl mathacrylate (bis-GMA) monomers, glass ionomer sealants are composed of fluoroaluminosilicate glass powder and an aqueous-based polyacrylic acid solution [10,20]. The most prominent advantage of resin-based sealing materials is their good durability, while glass ionomer sealants show advantageous fluoride-releasing properties.

The combination of the advantages of the two aforementioned materials was the aim of further materials. For example, compomers are resin-based materials with additional fluoride releasing properties, while resin-modified glass ionomers are glass ionomer sealants with additional resin components [10,40,41]. Older methods to protect against caries in the pits and fissure system include sealing with zinc phosphate cement, mechanical fissure eradication, prophylactic odontotomy, or chemical treatment with silver nitrate [10]. However, these methods are no longer routinely used thanks to the more convincing effects of sealing materials on the basis of resin or glass ionomer cement.

However, resin-based sealants and glass ionomer sealants also have disadvantages when they are used as sealing materials. In terms of resin-based materials for sealing, one disadvantage includes polymerization shrinkage, potentially resulting in microleakage, which allows saliva and bacteria to penetrate the occlusal barrier [42,43]. Furthermore, a stronger biofilm accumulation seems to occur on resin-based materials [15]. In cases when glass ionomer cements are used for sealing fissures and pits, fractures of the material can occur due to its reduced ability to withstand occlusal forces [10]. However, the most important issue when placing sealing materials is the capability of adhesion of the single material to the hard substance of the tooth. Sealing materials are only effective in preventing caries when they perfectly adhere to the tooth surface.

Methods to improve the retention of the sealing material on the tooth surface are, inter alia, a thorough cleaning of the occlusal surface with, for example, hydrogen peroxide, pumice, as well as air abrasion and pretreatment with acid [44]. With regard to the retention and long-term success of the sealing materials, numerous studies exist which compare different materials. A Cochrane review from 2013 also considered the effectiveness of different sealing materials. However, its authors did not come to a definite conclusion regarding the reduction of caries due to the use of a particular material for sealing pits and fissures [10,20]. Albeit, the results of various studies have shown that a reduced caries frequency is associated with an improved retention. Another Cochrane review from 2017 also stated that comparisons between glass ionomer to resin sealants remained inconclusive [45]. However, there is a clear advantage of resin-based sealing versus fluoridation, whereas no difference could be found between glass ionomer sealing and fluoridation [16]. This could be seen as an indirect suggestion for favoring resin-based sealing materials. Nevertheless, if it is impossible to isolate the teeth, for example, in the case of incompliant patients, children, or teeth in eruption, glass ionomer sealing materials should be preferred [46].

**Figure 1:**
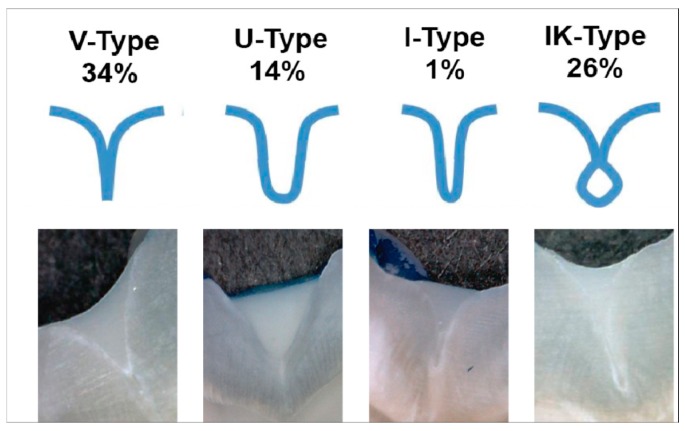
Different shapes of fissures.

Nevertheless, a positive correlation between the good retention of the sealing material and the occurrence of caries seems to be undeniable. Critical for the successful retention of the sealing material is a successful penetration of the material into the pits and fissures. Interestingly, effective penetration of a resin-based sealing material is independent of the specific material used. However, the morphology of the fissures significantly influences the penetration of the sealing material [47]. Although “Y”-shaped fissures presented a low penetration of the sealing materials, a “U” or “V” shape allowed for good penetration of the sealant [47]. Differences of the fissure morphologies are visualized in Figure 1. Long-term success of the resin-based materials might also be influenced by the use of a bonding system [10,48,49] and various pretreatment strategies such as laser irradiation [50], air abrasion of the tooth surface [51], or preheating of the sealing material [52].

## 4. Sealing of Non-Cavitated Lesions

Independent of attempts to extend the longevity of the sealants, the sealing of pit and fissures can generally be recommended. Because the emergence of caries has changed in the last decades, not only teeth in eruption should be sealed [10]. The application of a sealing material should be based on personal, tooth-, and surface- risk, which can change during the patient’s life [10]. Risk-based sealing seems to be an ideal approach for patients with a differing caries risk, while routinely sealing of primary molars has been shown to be effective in patients with equal caries risk [53]. However, no superior technique exists to forecast tooth decay [54]; only caries experience can function as a prognostic tool to forecast tooth decay [55]. In addition to the use of sealing materials for primary prevention for the avoidance of caries occurrence, a secondary prevention in areas already affected by caries should also be considered [10]. Arresting the caries and eliminating viable microorganism under the sealing material is the purpose of this application [10].

**Figure 2:**
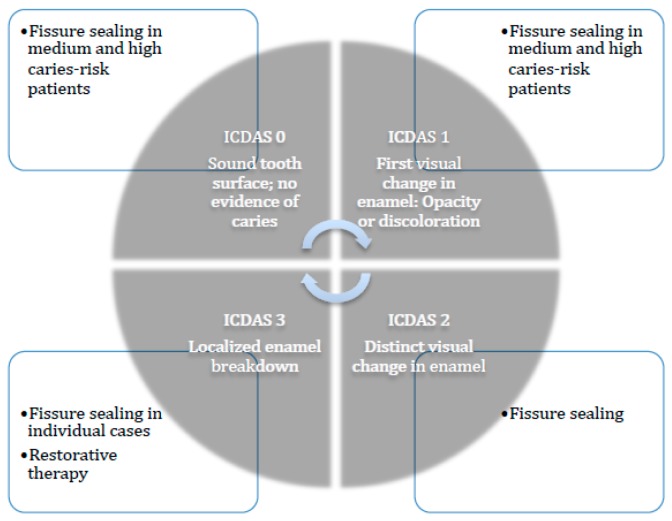
Treatment options depending on the ICDAS (International Caries Detection and Assessment System) codes.

As soon as a cavity is identified, conventional restorative methods must be implemented. In case of an intact enamel layer—a so-called non-cavitated lesion [56]—the application of a sealing material should be considered [57]. This method arrests the progression of this hidden caries and therefore conserves the tooth structure by means of delaying and minimizing operative procedures [58,59]. A systematic review on this topic reported that sealing with resin-based materials arrested the progression of carious lesions [59,60,61,62]; however, sealing with glass ionomer cement did not arrest caries progression [63]. These results are based on the intact and tight closure of the sealing material which did not occur when glass ionomer cement was used [57]. Further studies have shown positive effects of sealing non-cavitated lesions as long as the sealant is intact [59,60]. Fissure sealing was also successful in arresting caries in cases where the caries had already penetrated the dentin. However, the prerequisite was a tight connection between sealing material and tooth surface [64]. The use of sealing materials in carious deciduous teeth was also successful in so far as this technique was not inferior to the invasive techniques [59,61,62]. A decision aide for the application of fissure sealants is given in Figure 2.

## 5. Follow-Up Treatment

Because the long-term success of pit and fissure sealing depends on the intact mechanical barrier of the material, regular control is essential. This applies to the application of sealing materials in the context of primary prevention and even more in the context of secondary prevention. Therefore, strict compliance from the patient and/or the patient’s parents as well as access to regular recall appointments is a sine qua non [61,65]. Otherwise, saliva and food remnants can penetrate the leaked sealing material sustaining bacterial and biofilm growth with the result of caries development or progression beneath the sealing material [61].

Apart from the aforementioned negative effects if the sealing material is leaking, studies have examined possible side effects. Patients participating in clinical trials on sealing materials did not show any adverse events [26,27,66]. Nevertheless, reports exist about possible oestrogen-like effects of resin-based materials containing bisphenol A, such as bis-GMA or bis-DMA. Bisphenol A was detected in the saliva of patients for up to three hours after the application of resin-based sealing material [67,68,69]. Nevertheless, studies have concluded that patients are not at risk for oestrogen-like effects after the application of pit and fissure sealings [20,70].

## 6. Conclusions

In summary, clinical recommendations for the use of pit and fissure sealants are beneficial. The main recommendations are that sealing pit and fissures of primary and permanent teeth is safe and effective both in preventing and in arresting caries. However, the long-term success is dependent on regular checkups and the renewal of the sealing if required.
